# A CMOS Voltage Reference with PTAT Current Using DIBL Compensation for Low Line Sensitivity

**DOI:** 10.3390/s25216794

**Published:** 2025-11-06

**Authors:** Minji Jung, Youngwoo Ji

**Affiliations:** Department of Electronic Engineering, Hanbat National University, Daejeon 34158, Republic of Korea; 30242766@edu.hanbat.ac.kr

**Keywords:** low-power, PTAT current, CMOS voltage reference, line sensitivity, DIBL effect, temperature coefficient

## Abstract

This paper presents a low-power CMOS voltage reference with low supply sensitivity, designed and verified in a 180 nm standard CMOS technology. A DIBL-based line-sensitivity (LS) compensation path is incorporated into the conventional PTAT generation circuit to simultaneously provide a reference voltage and a bias current with improved LS. The proposed circuit achieves LS values of 0.01%/V for the voltage reference and 0.07%/V for the bias current reference over a supply voltage range of 1.4 V to 2 V. It generates a reference voltage of 538 mV and a PTAT current of 38 nA, consuming 68 nW. The simulated temperature coefficient is 58 ppm/℃ from −40 °C to 130 °C, and the power supply rejection ratio is −59 dB at 100 Hz.

## 1. Introduction

With the rapid development of sensor technology, the demand for energy-efficient systems such as the Internet of Things (IoT) and self-powered wearable devices has been steadily increasing. In these energy-constrained systems, the power and area limitations of integrated circuits (ICs) become critical design considerations. Given the limited energy budget, ICs must minimize power consumption while preserving the key performance metrics. Typically, sensor-based systems rely on constrained energy sources such as miniature batteries or energy harvesters. Due to the limited driving capability, the supply voltage of core ICs becomes highly susceptible to fluctuations induced by power-intensive events such as wireless power/data transmission or sensing operations. In addition, duty-cycled operation, which is commonly used to maximize energy efficiency, introduces abrupt supply transients whenever the system transitions from sleep to active mode. These challenges highlight the necessity of reliable voltage performance under dynamic supply conditions.

Voltage reference and bias current generator are fundamental building blocks for analog and mixed signal ICs, especially in sensor applications, where they directly affect performance and accuracy of the entire system, providing well-defined outputs to ensure reliability against process, voltage and temperature (PVT) variations. For voltage references, a bandgap reference (BGR) [1,2] has been widely adopted since BJTs exhibit strong immunity to PVT variations. To mitigate the temperature coefficient (TC) of the BJT, the BGR adds a proportional-to-absolute-temperature (PTAT) voltage to the complementary-to-absolute-temperature (CTAT) voltage derived from the BJT. However, BGRs require a sufficient current to alleviate the effect of the saturation current, which limits further power reduction. As an alternative, CMOS references [3,4,5,6] can minimize the power consumption by operating the transistors in the subthreshold region. Since CMOS references show relatively higher supply dependency than BGRs, several approaches have been proposed to suppress it, including DIBL-based compensation [7], self-biasing loop [8], double regulation [9], pre-regulator [10], self-cascode [11] and amplifier-based loop [12]. These techniques achieve line sensitivities (LS) as low as 0.003~0.3%/V. However, the conducting current with exponential temperature dependency restricts direct use as bias currents, eventually necessitating an additional stage to separately generate bias currents. On the other hand, CMOS references employing a PTAT current can provide both a reference voltage and bias current at the same time [4,13,14,15]. However, their LS is relatively higher compared to LS-improved designs, and the LS of the bias current has not been reported.

This paper presents a CMOS voltage reference that generates PTAT currents in each branch while applying DIBL-based LS compensation to both reference voltage and bias current simultaneously. This work achieves both low-power operation and low LS in voltage and current while maintaining a stable TC. The proposed circuit is designed in a 180 nm standard CMOS technology, achieving LS values of 0.01%/V for reference voltage and 0.07%/V for bias current, which is improved by >10x compared with a conventional PTAT current generator under the same conditions. The TC of the reference voltage is 58 ppm/℃ over a temperature range from −40 °C to 130 °C. The PSRR at 100 Hz is −59 dB without using any decoupling capacitor.

The remainder of this paper is structured as follows. Section 2 introduces the proposed reference generator applied with the DIBL effect and the detailed derivation process to determine the design parameters to improve the LS. Section 3 presents the simulation results of the proposed circuit. Section 4 concludes the paper.

## 2. Proposed Voltage Reference with PTAT Current Using DIBL Compensation

### Design Description

For low-power implementation, CMOS transistors can be operated in the subthreshold region, where the conducting current is governed by

(1) $$\;\;\;\;\;\;\;\;\;\;\;I=I_{0}\cdot \frac{W}{L}\cdot \exp\left(\frac{V_{GS}-V_{th}}{mV_{T}}\right)\left(1-\exp\left(-\frac{V_{DS}}{V_{T}}\right)\right),$$

where $I_{0}=\mu C_{OX}\left(m-1\right)V_{T}^{2}$ and μ, $C_{OX}$, *m* and $V_{T}\;$ denote the carrier mobility, gate capacitance, subthreshold slope factor and thermal voltage, respectively. The exponential term in the last part of Equation (1) becomes negligible when $V_{DS}\;$> 4$V_{T}$. In addition, to account for the finite output resistance, the drain-to-source voltage ($V_{DS}$) dependency [16,17,18] should be considered, and the current Equation can be rearranged as follows:

(2) $$I≅I_{0}\cdot \frac{W}{L}\cdot \exp\left(\frac{V_{GS}-V_{th}+\eta V_{DS}}{mV_{T}}\right),$$

where η is a device parameter to model the effect of drain-induced barrier lowering (DIBL). As the channel length of a MOSFET decreases, η increases, revealing higher $V_{DS}$ dependency [7,19]. Table 1 summarizes exemplary values of η as a function of channel length, which will be used as a cornerstone for achieving LS suppression. Since the DIBL-induced term is dominated by the first-order component and $\frac{\eta V_{DS}}{mV_{T}}$ is much smaller than 1, Equation (2) can be approximated by first-order Taylor expansion as [17]:

(3) $$\;I=I_{0}\cdot \frac{W}{L}\cdot \left(1+\frac{\eta V_{DS}}{mV_{T}}\right)\cdot \exp\left(\frac{V_{GS}-V_{th}}{mV_{T}}\right).$$

**Figure 1:**
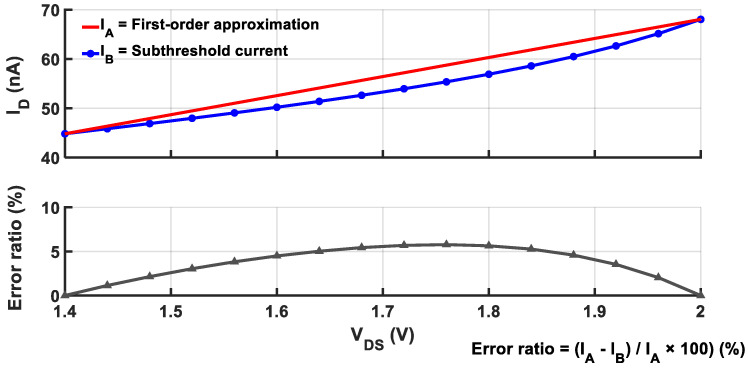
V_DS_ dependency of the subthreshold current and its first-order approximation.

Figure 1 compares the subthreshold current with the linear approximation model, showing the worst-case relative error is ~5% when the minimum length in the technology is used. Table 2 quantifies the fractional contribution of each order term and reveals that the first-order term provides the major contribution.

**Figure 2:**
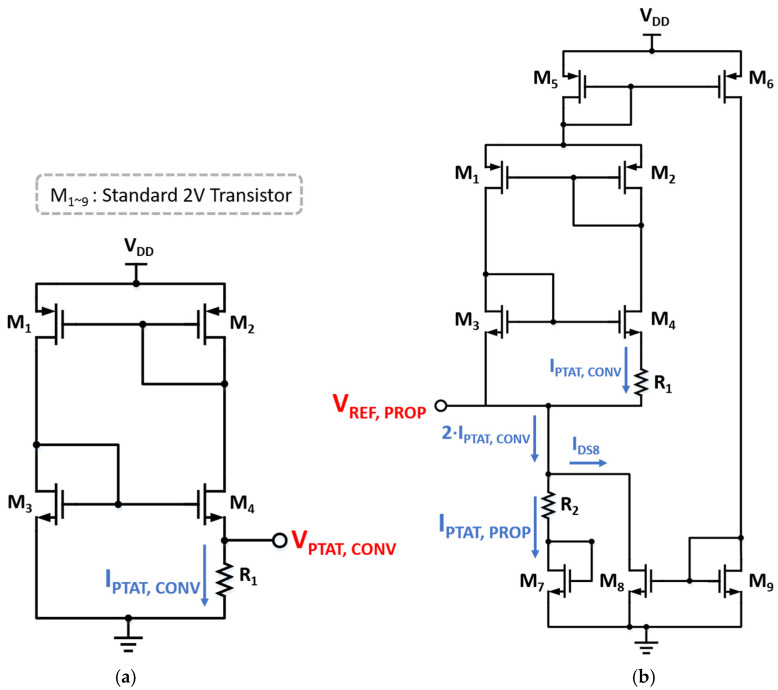
(**a**) Conventional PTAT current generator and (**b**) proposed voltage reference with PTAT current including DIBL compensation.

Figure 2a illustrates the conventional PTAT current generator, which is composed of two PMOS transistors (M1 and M2) for current regulation, two NMOS transistors (M3 and M4) for PTAT voltage generation, and a resistor for voltage-to-current conversion. The bodies of all transistors are connected to their sources.

Assuming that *L_3_* = *L_4_* and *I_0,3_* = *I_0,4_* in Equation (1), the resulting PTAT voltage and current can be written as

(4) $$V_{PTAT,CONV}=V_{GS,3}-V_{GS,4}=mV_{T}\ln\left(\frac{W_{4}}{W_{3}}\right),$$

(5) $$I_{PTAT,CONV}=\frac{V_{PTAT,CONV}}{R_{1}}=\frac{mV_{T}\ln\left(\frac{W_{4}}{W_{3}}\right)}{R_{1}}.$$

In the conventional PTAT current generator, because M4 exhibits a much larger output resistance ($r_{o}$) than that of the diode-connected M2 ($\frac{1}{g_{m}}$), the drain voltage of M4 varies linearly with the supply voltage, resulting in the LS in the right branch to be determined by M4. By applying the model of the *V_DS_* dependency in Equation (3), Equation (5) can be modified to include the LS effect as

(6) $$\;I_{PTAT,CONV}≅\frac{mV_{T}\ln\left(\frac{W_{4}}{W_{3}}\left(1+\frac{\eta_{4}V_{DD}}{mV_{T}}\right)\right)}{R_{1}},$$

where $\eta_{4}$ is the DIBL parameter of M4. The LS can be derived by differentiating Equation (6) on $V_{DD}$, yielding

(7) $$LS_{CONV}=\frac{\partial I_{PTAT,CONV}}{\partial V_{DD}}=\frac{1}{R_{1}}\cdot \frac{\eta_{4}}{1+\frac{\eta_{4}V_{DD}}{mV_{T}}}.$$

Equation (7) can be further approximated as $\eta_{4}/R_{1}$ for sufficiently long-channel devices where $\eta_{4}$ is small.

Figure 2b describes the proposed reference voltage with PTAT current generator. It preserves the conventional PTAT core while adding a CMOS transistor (M7) and a resistor ($R_{2}$) in the pull-down path. Since the flowing current is the same as Equation (5), the PTAT current ($I_{PTAT,\;PROP}$) of M7 and the reference voltage ($V_{REF,PROP}$) are given by

(8) $$I_{PTAT,PROP}≅\frac{2mV_{T}\ln\left(\frac{W_{4}}{W_{3}}\right)}{R_{1}},$$

(9) $$V_{REF,PROP}=V_{GS,7}+I_{PTAT,PROP}R_{2}=V_{th7}-mV_{T}\ln\left(\frac{I_{0,7}}{I_{PTAT,PROP}}\cdot \frac{W_{7}}{L_{7}}\right)+I_{PTAT,PROP}\cdot R_{2}.$$

In Equation (9), the threshold voltage and the logarithmic term act as CTAT voltages, while the last term becomes a PTAT voltage. Therefore, the TC of $V_{REF,PROP}$ can be minimized by adjusting $R_{2}$. To improve the LS, a DIBL-based compensation path is introduced in the right branch, which consists of M5, M6, M8 and M9. M5 and M6, with shorter length and larger η than other transistors, generate a current with higher supply dependency. The level of the current ($I_{DS8}$) is scaled by M8 and M9 configured as a current mirror. Then, the final PTAT current can be obtained by subtracting $I_{DS8}$ from $2I_{PTAT,CONV}$, resulting in the final $I_{PTAT,PROP}$ as

(10) $$\;I_{PTAT,PROP}=2I_{PTAT,CONV}-I_{DS8}=2\cdot I_{PTAT,CONV}\cdot \left(1-\left(\frac{W_{6}}{W_{5}}\cdot \frac{W_{8}}{W_{9}}\right)\right).$$

In Equation (10), the DIBL effect of $I_{PTAT,CONV}$ can be incorporated using Equation (6), while that of $I_{DS8}$ can be modeled by changing *W_6_*, since the drain voltage of M6 rarely varies with the supply voltage and M6 becomes the dominant contributor to LS in the compensation path. Substituting the *V_DS_*-dependency in Equation (3) and $I_{PTAT,CONV}$, Equation (10) can be rewritten as

(11) $$\;I_{PTAT,PROP}≅\frac{2mV_{T}\ln\left(\frac{W_{4}}{W_{3}}\left(1+\frac{\eta_{4}V_{DD}}{mV_{T}}\right)\right)}{R_{1}}\cdot \left(1-\frac{W_{6}}{W_{5}}\cdot \frac{W_{8}}{W_{9}}\left(1+\frac{\eta_{6}V_{DD}}{mV_{T}}\right)\right).\;$$

Differentiating Equation (11) with respect to $V_{DD}$, the LS of the proposed circuit becomes

(12) $$\;LS_{PROP}=\frac{\partial I_{PTAT,PROP}}{\partial V_{DD}}=\frac{2}{R_{1}}\frac{\eta_{4}}{1+\frac{\eta_{4}V_{DD}}{mV_{T}}}\left(1-\frac{W_{6}}{W_{5}}\cdot \frac{W_{8}}{W_{9}}\left(1+\frac{\eta_{6}V_{DD}}{mV_{T}}\right)\right)-\frac{2}{R_{1}}\eta_{6}\frac{W_{6}}{W_{5}}\cdot \frac{W_{8}}{W_{9}}ln\left(\frac{W_{4}}{W_{3}}\left(1+\frac{\eta_{4}V_{DD}}{mV_{T}}\right)\right).$$

For low-power operation, $\frac{W_{6}}{W_{5}}$ and $\frac{W_{8}}{W_{9}}$ are designed to be sufficiently small so that the current in the compensation branch remains much smaller than that in the core. In addition, assuming a sufficient length for M4, Equation (12) can be simplified to

(13) $$LS_{PROP}=\frac{\partial I_{PTAT,PROP}}{\partial V_{DD}}≅\frac{2}{R_{1}}\eta_{4}-\frac{2}{R_{1}}\eta_{6}\frac{W_{6}}{W_{5}}\cdot \frac{W_{8}}{W_{9}}ln\left(\frac{W_{4}}{W_{3}}\right).$$

It should be noted that Equation (13) contains a negative term, which is derived from the LS compensation path. By setting Equation (13) equal to zero, the theoretical optimum condition for achieving zero LS can be derived as

(14) $$\frac{\eta_{4}}{\eta_{6}}=\frac{W_{6}}{W_{5}}\cdot \frac{W_{8}}{W_{9}}ln\left(\frac{W_{4}}{W_{3}}\right).$$

The overall design procedure can be summarized as follows:

1. Core PTAT design: Design the PTAT generator considering both current level and TC based on Equation (6), determining *W_3_*, *W_4_* and $\eta_{4}$.
2. Compensation path sizing: Determine the current level in the LS-improvement paths, which defines *W_5_*, *W_6_*_,_ *W_8_* and *W_9_*.
3. Optimum length selection: Calculate the theoretical optimum value for $\eta_{6}$ by using Equation (14) and determine *L_6_* based on Table 1.
4. Final adjustment: Tune the design parameters in the LS-improvement paths.

**Figure 3:**
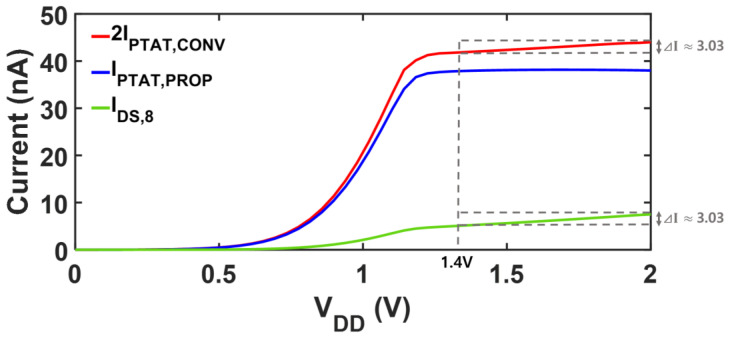
Simulated supply voltage dependency of each current for LS compensation.

The transistor dimensions and their operating currents are shown in Table 3. Figure 3 shows the LS of each current after applying the DIBL-based LS-compensation. When the supply voltage varies from 1.4 V to 2.0 V, $2I_{PTAT,CONV}$ increases by 3.03 nA, showing an LS of 9.56%/V. The compensation current, *I_DS8_*, exhibits the same variation amount with $2I_{PTAT,CONV}$, while the level *I_DS8_* is much smaller. Therefore, subtracting *I_DS8_* from $2I_{PTAT,CONV}$ yields the LS compensated current, $I_{PTAT,\;PROP}$, which achieves an LS of 0.07%/V. $I_{PTAT,\;PROP}$ can be tapped from M7 to bias other circuits. The residual variation in $I_{PTAT,\;PROP}$ results from the nonlinearities in the simplified modeling.

**Figure 4:**
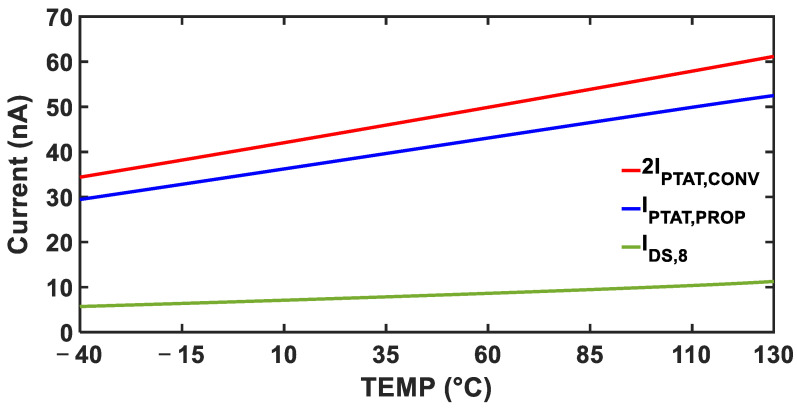
Simulated temperature dependency of each current.

Although the proposed DIBL-based LS-compensation diverts a portion of the core current and slightly reduces the level of current as in Equation (10), the temperature dependency of each current remains unaffected because all currents are just scaled versions of the original PTAT current in Equation (5), as shown in Figure 4.

**Figure 5:**
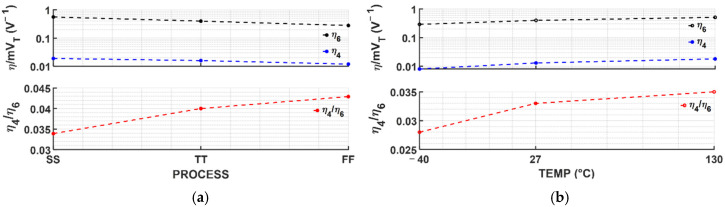
Simulated $\eta_{4}$, $\eta_{6}$ and $\frac{\eta_{4}}{\eta_{6}}$ depending on (**a**) process-skewed corners and (**b**) temperatures.

Although the theoretically optimum value for LS compensation is set by the dimension of the transistors as Equation (14), PVT variations affect $\eta_{4}$ and $\eta_{6}$. Figure 5 presents the dependency of $\eta_{4}$ and $\eta_{6}$ on process and temperature, respectively. Each of $\eta_{4}$ and $\eta_{6}$ varies by up to 70% across process-skew (Figure 5a); however, because they vary in the same direction, the variation in the ratio $\frac{\eta_{4}}{\eta_{6}}$ is reduced to ~20%. Similarly, in the case of temperatures (Figure 5b), each of $\eta_{4}$ and $\eta_{6}$ varies by up to about 55%, but, since their temperature variation follows the same trend, the variation in the ratio $\eta_{4}/\eta_{6}$ is reduced to ~20%. The worst case occurs at the fast-skewed process corner, resulting in LS values of 4.4%/V and 9.3%/V for $V_{REF,PROP}$ and $I_{PTAT,PROP}$, which is still lower than the typical case of a conventional PTAT current generator.

## 3. Post-Layout Simulation Results

**Figure 6:**
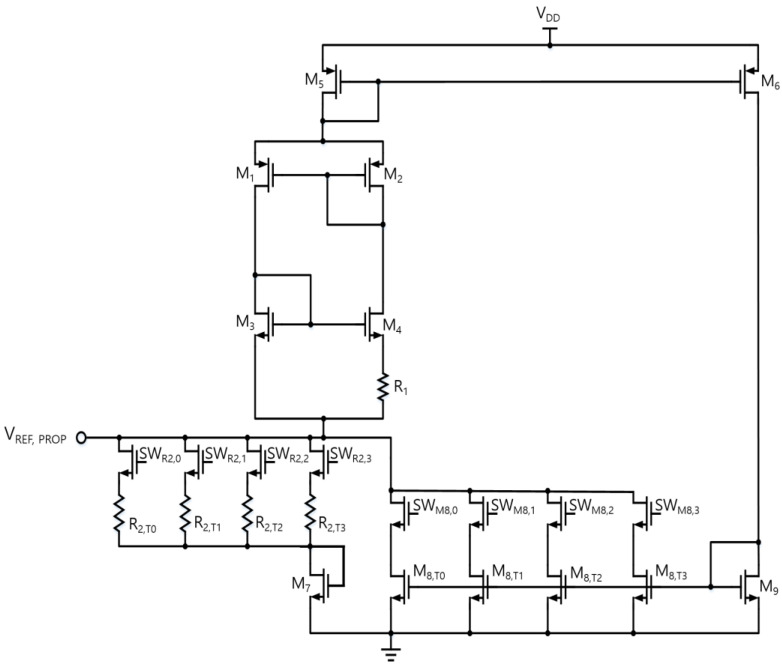
The proposed circuit diagram with trimming options for line sensitivity (M8) and temperature coefficient (R2).

**Figure 7:**
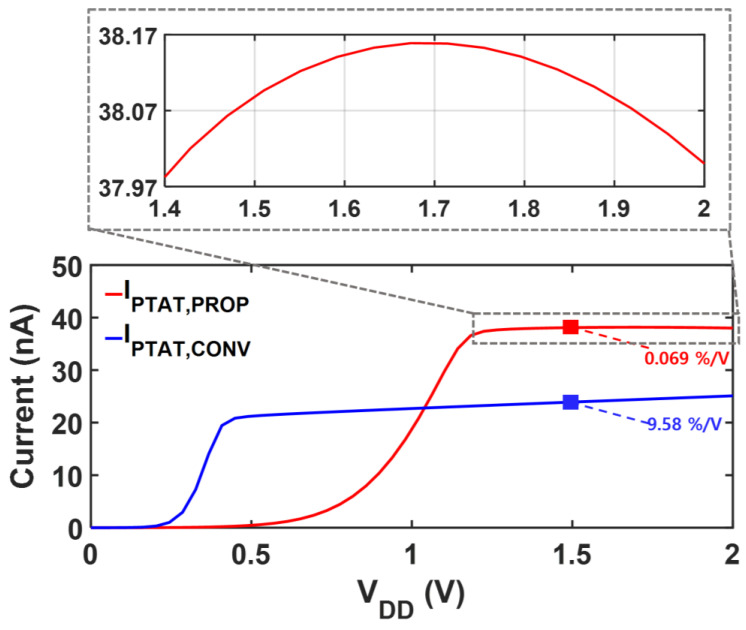
Simulated LS of $\;I_{PTAT,CONV}\;\mathrm{and}\;I_{\mathrm{PTAT},PROP}.$

**Figure 8:**
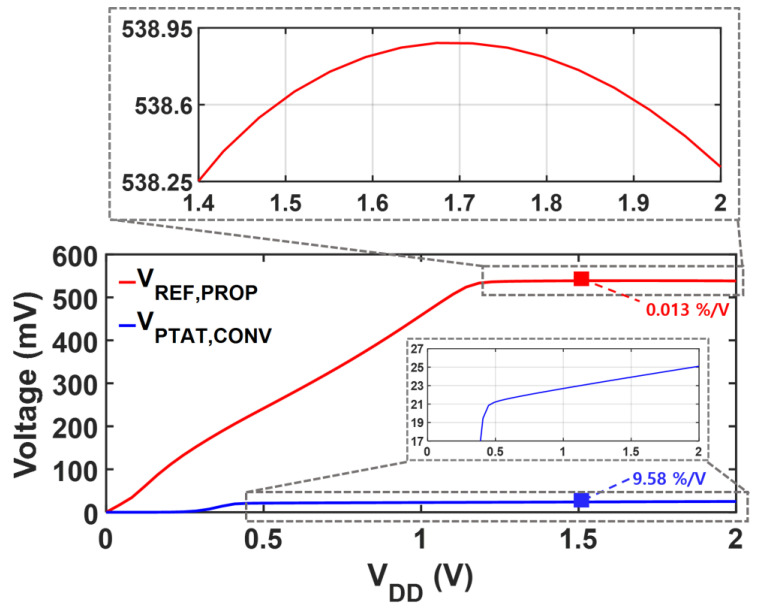
Simulated LS of $\;V_{PTAT,CONV}\;\mathrm{and}\;V_{REP,PROP}.$

Figure 6 shows the proposed reference circuit with trimming options to mitigate the potential effects of process and mismatch variations on the LS and TC. Each 4-bit trimming code independently adjusts $R_{2}$ for TC and M8 for LS. A higher code for $R_{2}$ makes both *V_REF,PROP_* and *I_PTAT,PROP_* more PTAT, whereas a higher code for M8 corresponds to a larger compensation current. Figure 7 compares the supply characteristics of the currents of the conventional PTAT current generator in Figure 2a and the proposed voltage reference with PTAT current using DIBL compensation in Figure 2b. Without LS-compensation, the LS of $I_{PTAT,CONV}\;$ follows Equation (7), exhibiting a LS of 9.56%/V over a supply range of 1 V to 2 V. By employing the same PTAT core with the additional LS-compensation path, the LS of $I_{PTAT,PROP}$ is significantly improved to 0.07%/V in the supply range from 1.4 V to 2 V. Figure 8 presents a comparison of each voltage against the supply voltage variation. The LS of $V_{REF,PROP}$ is suppressed to 0.01%/V across a supply voltage range of 1.4 V to 2 V, whereas that of $V_{PTAT,CONV}\;$ is 9.56%/V over 1 V to 2 V. The proposed DIBL compensation scheme achieves LS values of 0.01%/V and 0.07%/V for the reference voltage and PTAT current at the expense of a higher minimum supply voltage. The minimum supply voltage condition of the conventional circuit in Figure 2a is expressed as

(15) $$V_{DD,min,CONV}=V_{DS,1}+V_{GS,3},$$

while that of the proposed circuit (Figure 2b) becomes

(16) $$V_{DD,min,PROP}=V_{REF,PROP}+V_{DS,1}+V_{GS,3}+V_{GS,5}=V_{REF,PROP}+V_{DD,min,CONV}+V_{GS,5}.$$

**Figure 9:**
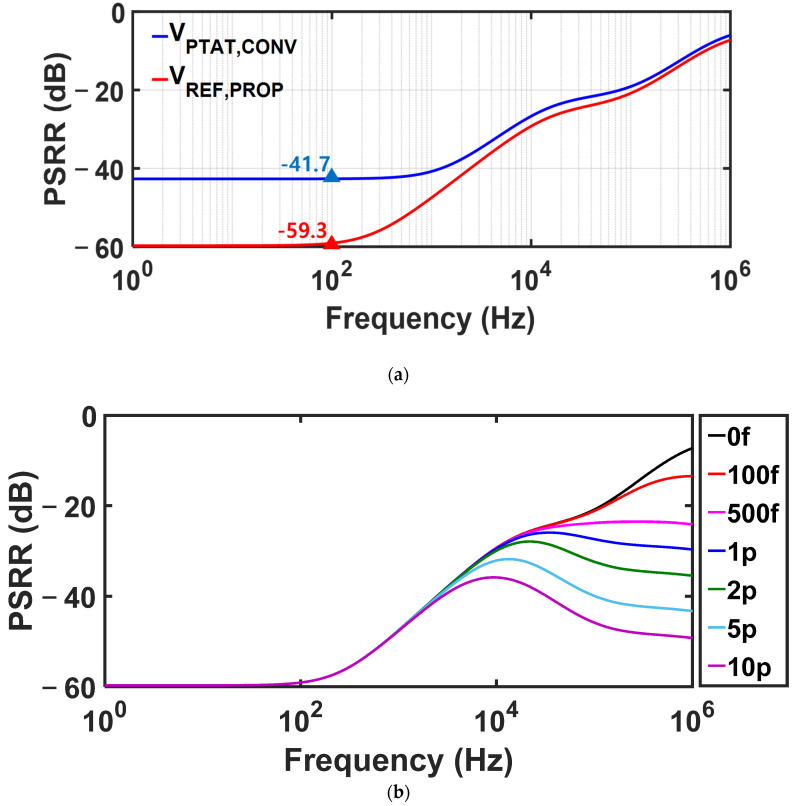
Simulated PSRR of $V_{REF,PROP}$ at a supply voltage of 1.4 V (**a**) without any decoupling capacitor and (**b**) with different sizes of load capacitors.

Figure 9a shows the simulated PSRR of the proposed circuit without any decoupling capacitor. At 100 Hz, the proposed circuit achieves a PSRR of −59 dB. Figure 9b depicts the improvement of high-frequency PSRR when a load capacitor is added to $V_{REF,PROP}$. 

**Figure 10:**
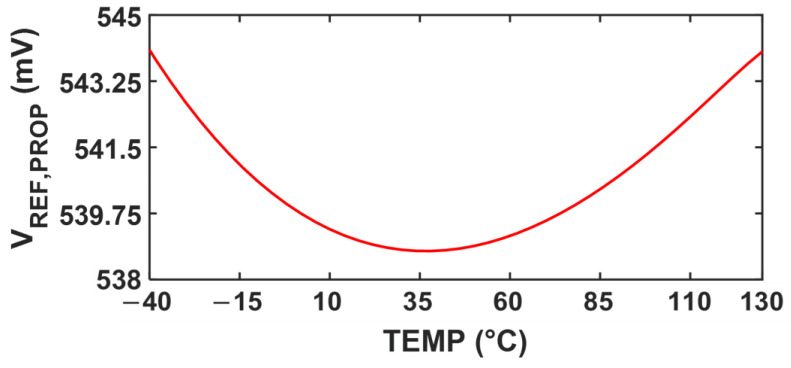
Simulated TC of $\;V_{REF,PROP}$.

**Figure 11:**
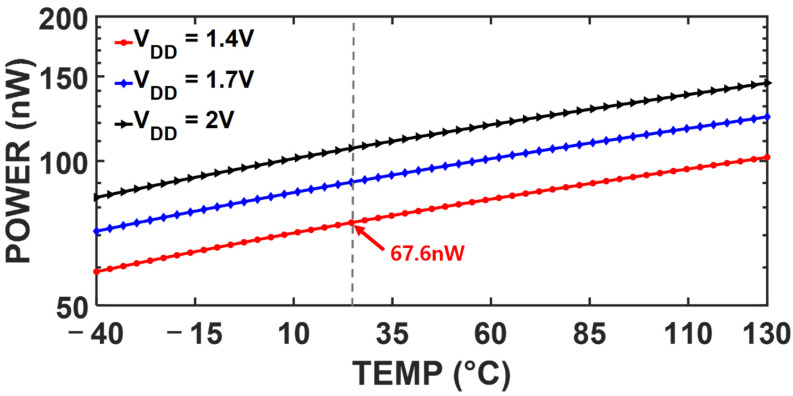
Simulated power consumption under different temperatures and supply voltages.

Figure 10 illustrates the temperature dependency of $V_{REF,PROP}$, indicating the TC of 58 ppm/℃  over the range −40 °C to 130 °C. The proposed LS-compensation scheme achieves low LS while maintaining the TC below 100 ppm/℃. Figure 11 shows the power consumption of the proposed reference generator as a function of temperature under different *V_DD_* levels. The proposed circuit consumes 68 nW with a supply voltage of 1.4 V at room temperature.

**Figure 12:**
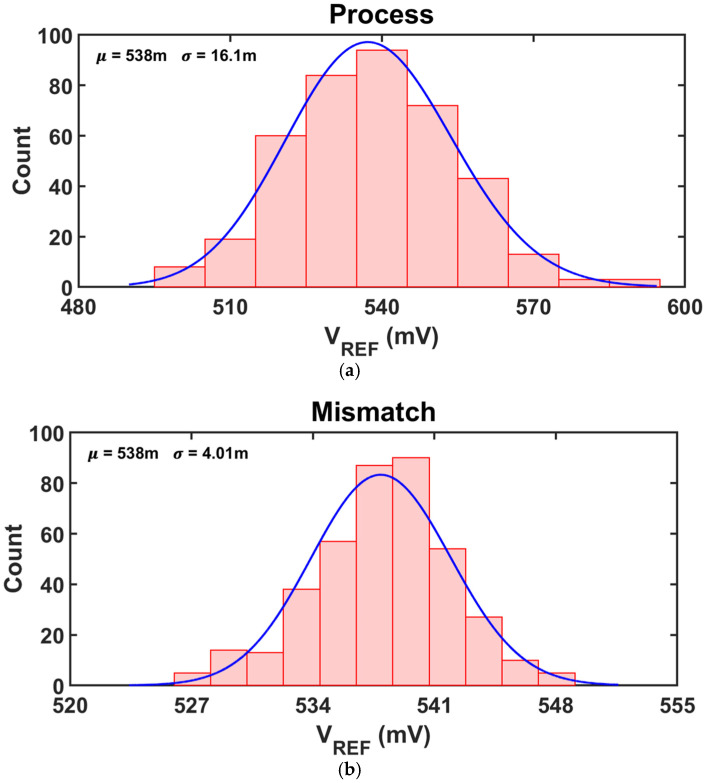

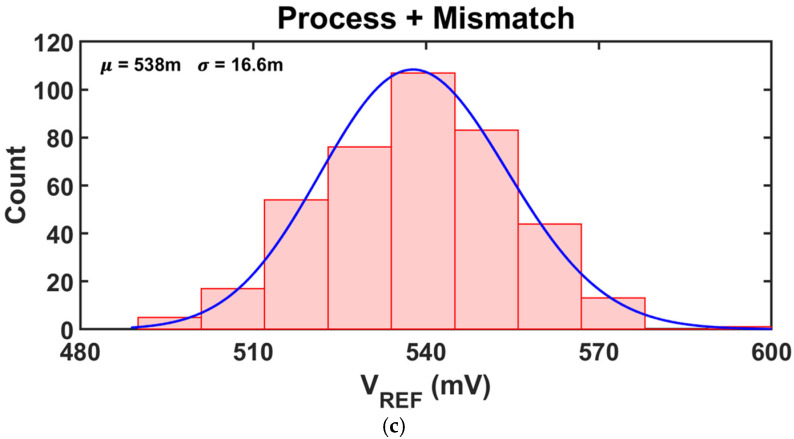
400 runs of Monte Carlo Simulation results of *V_REF,PROP_*. (**a**) process variations, (**b**) mismatch variations, (**c**) both.

Figure 12 presents the distributions of *V_REF,PROP_* under process, mismatch and both variations. The average *V_REF,PROP_* is about 538 mV regardless of the simulation conditions, while the standard deviation is mainly determined by the process variation, as shown in Figure 12a. When both process and mismatch variations are applied, the total *σ*/*µ* is 3.1%.

**Figure 13:**
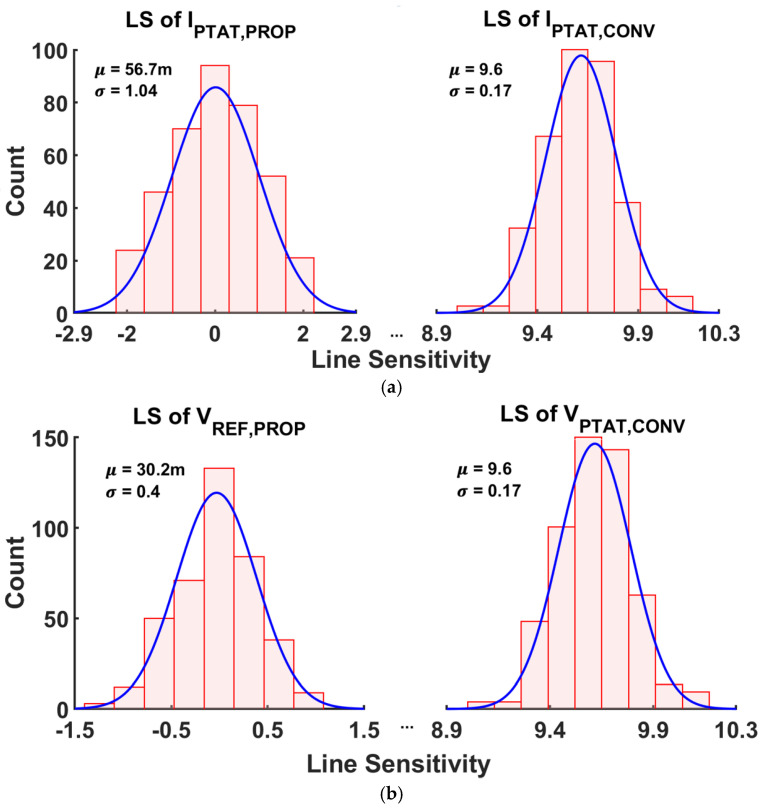
400 runs of Monte Carlo Simulation results for the LS of (**a**) PTAT current and (**b**) voltage reference.

The impact of device mismatch on LS was verified through 400 runs of Monte Carlo simulations (Figure 13). Figure 13a shows the distribution of LS of $I_{PTAT,PROP}$ before and after compensation in the same graph. Without compensation, $\;I_{PTAT,CONV}$ exhibits an average LS of 9.56%/V with a standard deviation of 0.17%/V. After applying the proposed LS-compensation, the average value is reduced to 0.06%/V, with a standard deviation of 1.04%. Figure 13b shows the distribution of the LS of $V_{REF,PROP}$ before and after compensation. The average LS improves from 9.56%/V without compensation to 0.03%/V with compensation. The worst-case LS after compensation is even smaller than the best-case LS without compensation in both current and voltage cases, validating the proposed scheme.

**Figure 14:**
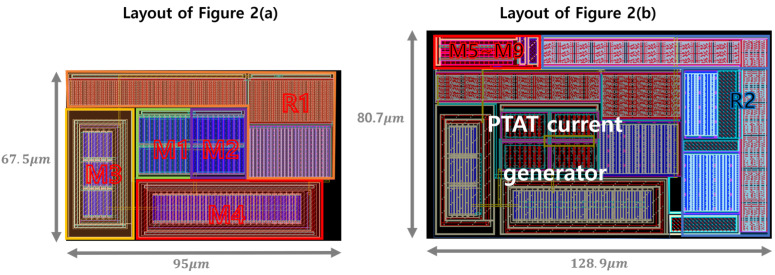
Layout of (**a**) the conventional PTAT current generator (Figure 2a) and (**b**) the proposed voltage reference with PTAT current (Figure 2b).

Figure 14 demonstrates the exemplary layouts of the conventional PTAT current generator and proposed voltage reference with PTAT current including DIBL compensation in Figure 2. The active areas of the layouts are 6412$\;\mu m^{2}$ (67.5 μm × 95 μm) and 10,402 $\mu m^{2}$ (80.7 μm× 128.9 μm), respectively. The proposed reference generator achieves low LS with only ~4000$\;\mu m^{2}$ of additional active area.

**Figure 15:**
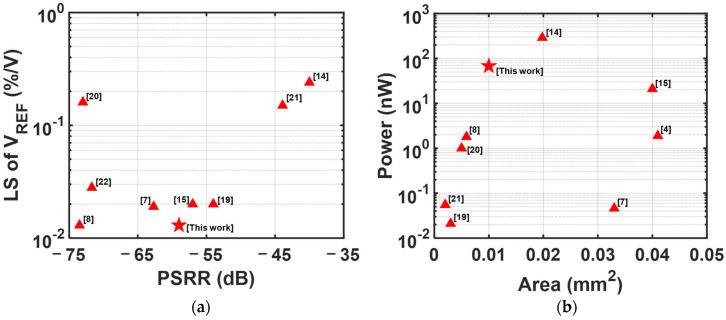
Benchmark against recently reported voltage references in Table 3. (**a**) PSRR versus LS of the voltage reference and (**b**) Power and area.

Table 4 and Table 5 summarize the performance of the proposed circuit and compare it with recently reported designs according to the output type of voltage and bias current, respectively. Figure 15 benchmarks the proposed circuit with recent works in terms of reference voltage. Figure 15a shows the LS of the reference voltage versus its PSRR, and Figure 15b provides a benchmark comparing the power against the area.

**Figure 16:**
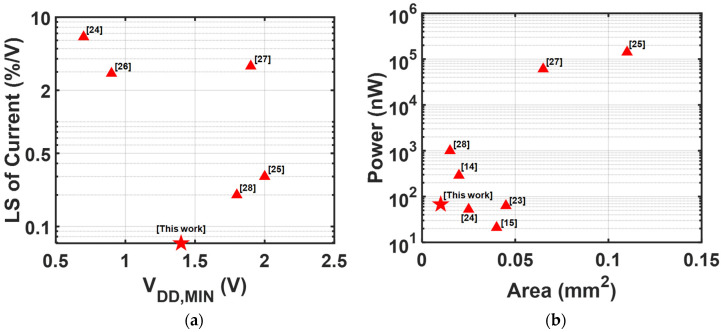
Benchmark against recently reported bias current generators in Table 4. (**a**) LS of the current and minimum supply voltage, and (**b**) Power and area.

Figure 16 compares the performances of the previously reported current generator, which can be used as a bias current, by visualizing the LS and minimum supply voltage (Figure 16a) and power against area (Figure 16b). The proposed circuit achieves superior line sensitivity performance compared to conventional bias current references while minimizing the power-area overhead.

## 4. Conclusions

This paper presents a CMOS voltage reference with a PTAT current employing DIBL-based LS-compensation. The proposed design achieves both low-power operation and low LS in voltage and current while maintaining a stable TC. The circuit was designed and simulated in a 180 nm standard CMOS technology, generating a reference voltage of 538 mV and a PTAT current of 38 nA, consuming only 68 nW. The LS values of current and voltage outputs are 0.07%/V and 0.01%/V, respectively, within a supply voltage range of 1.4 V to 2 V. The circuit exhibits a simulated TC of 58 ppm/℃ across −40 °C to 130 °C,  and the PSRR of −59 dB at 100 Hz. The proposed voltage reference with a PTAT bias current provides both stable voltage and current in a single circuit and is well-suited for energy-constrained applications such as battery-powered electronics and IoT sensor systems due to its low supply sensitivity and low power consumption characteristics, enabling the system to function under dynamic supply conditions.

## Figures and Tables

**Table 1:** Exemplary values of η as a function of the length of the transistor PMOS.

Length (μm)	0.18	0.5	2	5	10	20
$\frac{\eta }{mV_{T}}$ ($V^{-1})$	0.4	0.11	0.061	0.028	0.016	0.00156

**Table 2:** Relative magnitudes of each expansion order.

order	1$\;\left(x\right)$	2$\;\left(\frac{x^{2}}{2!}\right)$	3$\;\left(\frac{x^{3}}{3!}\right)$
value	$3.7\times 10^{-3}$	$6.85\times 10^{-6}$	$8.44\times 10^{-9}$

**Table 3:** Transistor dimensions of the proposed circuit.

Transistor	Width (μm)	Length (μm)	Current (nA)
M1 = M2	1	10	21.7
M3	15	10	21.7
M4	25	10	21.7
M5	60	0.18	43.4
M6	4	0.18	4.8
M7	0.3	14	38.1
M8	3.2	10	5.4
M9	1.9	10	4.8

**Table 4:** Performance comparison with previously reported voltage references.

	This Work	[4]	[7]	[8]	[14]	[15]	[19]	[20]	[21]	[22]
Year	2025	2014	2020	2021	2015	2018	2023	2019	2017	2022
Technology (nm)	180	180	180	180	65	180	180	180	180	40
V_DD_ (V)	1.4~2	0.7~2.5	0.34~1.8	0.9~1.8	0.75~1.2	0.7~2	0.6~1.8	0.4~1.8	0.45~3.3	1.2~1.8
V_REF_ (V)	0.54	0.44	0.15	0.26	0.47	0.55	0.31	0.26	0.23	0.8
Temp. Range (°C)	−40~130	−20~85	0~100	−40~130	−40~90	−40~110	−20~80	−40~130	0~120	−40~90
TC (ppm/°C)	58	22.1	14.8	62	40	38	25	89.8	104	3
LS of V_REF_ (%/V)	0.01	0.057	0.019	0.013	0.24	0.02	0.02	0.16	0.15	0.028
PSRR (dB) @Freq. (Hz)	−59 @100	-	−62.7 @10	−73.5 @100	−40 @100	−57 @100	−54 @100	−73 @10	−43.9 @100	−71.7 @100
Power (nW)	67.6	1.9	0.046	1.8	290	21	0.021	1	0.055	9600
Area (mm^2^)	0.01	0.041	0.0332	0.0059	0.0198	0.04	0.003	0.005	0.002	-

**Table 5:** Performance comparison for output current of previously reported works.

	This Work	[14]	[15]	[23]	[24]	[25]	[26]	[27]	[28]
Year	2025	2015	2018	2006	2013	2023	2016	2017	2010
Technology (nm)	180	65	180	350	180	180	40	350	350
V_DD_ (V)	1.4~2	0.75~1.2	0.7~2	0.9~4	0.7~1.8	2~3.3	0.9~1.5	1.9~3.6	1.8~3
Current Type	PTAT	PTAT	PTAT	PTAT *	REF	REF	REF	REF	REF
Level of current at 27 °C (nA)	38	-	31	40	6	8500	96.5	16,000	96
LS of Current (%/V)	0.07	-	-	-	6.47	0.3	2.9	3.4	0.2
Temp. Range (℃)	−40~130	−40~90	−40~110	0~80	−40~120	−35~125	−40~125	−30~100	0~80
Power (nW)	67.6	290	21	63	52.5	142000	304	60800	1000
Area (mm^2^)	0.01	0.0198	0.04	0.045	0.025	0.11	-	0.065	0.015

* non-linear increased temperature compensated current.

## Data Availability

No new data were created or analyzed in this study. Data sharing is not applicable to this article.
